# Particle Therapy for Non-Small Cell Lung Tumors: Where Do We Stand? A Systematic Review of the Literature

**DOI:** 10.3389/fonc.2014.00292

**Published:** 2014-10-29

**Authors:** Krista C. J. Wink, Erik Roelofs, Timothy Solberg, Liyong Lin, Charles B. Simone, Annika Jakobi, Christian Richter, Philippe Lambin, Esther G. C. Troost

**Affiliations:** ^1^Department of Radiation Oncology (MAASTRO Clinic), GROW – School for Oncology and Developmental Biology, Maastricht University Medical Centre, Maastricht, Netherlands; ^2^Department of Radiation Oncology, Hospital of the University of Pennsylvania, Philadelphia, PA, USA; ^3^OncoRay – National Center for Radiation Research in Oncology, Faculty of Medicine and University Hospital Carl Gustav Carus, Technische Universität Dresden and Helmholtz-Zentrum Dresden-Rossendorf, Dresden, Germany; ^4^German Cancer Consortium (DKTK) Dresden and German Cancer Research Center (DKFZ), Heidelberg, Germany

**Keywords:** radiotherapy, NSCLC, photon, proton, C-ion, PSPT, PBS, IMPT

## Abstract

This review article provides a systematic overview of the currently available evidence on the clinical effectiveness of particle therapy for the treatment of non-small cell lung cancer and summarizes findings of *in silico* comparative planning studies. Furthermore, technical issues and dosimetric uncertainties with respect to thoracic particle therapy are discussed.

## Introduction

Over the last few decades, improvements in surgical techniques and (chemo)radiotherapy have increased the survival rate in patients with lung cancer (1). Despite this, the survival of patients with locally advanced non-small cell lung cancer (NSCLC) remains poor, with a 5-year overall survival (OS) of 15% for stage III NSCLC patients treated with concurrent chemoradiotherapy (2).

Prior studies have suggested that a higher tumor dose can result in increased locoregional control, and consequently in an improved disease-specific survival (3, 4). Different strategies to escalate the dose to the primary target volume have been studied, i.e., isotoxic dose enhancement, hyperfractionation, or dose-rescaling on an individual basis (5–7). However, the tumor-surrounding healthy tissues (healthy lung, heart, mediastinal structures, spinal cord, and brachial plexus) are often dose limiting.

Even though modern radiotherapy techniques (e.g., 3D conformal radiotherapy; 3D-CRT, or intensity-modulated radiation therapy; IMRT) decrease dose to surrounding tissues compared with 2D techniques, particle beam therapy may offer an even greater advantage. The favorable properties of charged particles in terms of dose distribution and radiobiological effectiveness make it an option worth investigating for the treatment of NSCLC. Treatment with protons or carbon ions can enable dose escalation beyond what can be achieved with the different photon techniques (8). There are, however, several difficulties with the delivery of particle beams to a tumor in the lung. These include the fact that the tumor and other anatomy may be moving, that variations exist in the water equivalent thickness of the beam path, and that the range uncertainties associated with pencil beam scanning (PBS) techniques are particularly sensitive to motion and setup errors (9, 10).

The aim of this article is to systematically review the currently available evidence on the clinical effectiveness and *in silico* comparative planning studies of particle therapy for the treatment of NSCLC. The technical issues and dosimetric uncertainties with respect to thoracic particle therapy are also discussed.

## Methods

The PubMed database was searched by two researchers to identify studies about particle therapy for NSCLC. Search items were lung, proton, particle, hadron, carbon, cancer, tumor, neoplasm, therapy, treatment, radiation therapy, radiotherapy, irradiation, and NSCLC. The search terms were used in different combinations and plural forms, and the search was limited to articles in English. References were screened for additional articles. Three types of studies were included: studies reporting on the outcomes of particle therapy for NSCLC, studies comparing dose distributions in (in silico) planning studies, and studies reporting on technical issues with particle therapy for NSCLC.

## Results

### Clinical outcome

All the studies reporting on clinical outcome of particle therapy for NSCLC were published by one of the following centers treating NSCLC patients with particle beams: the Loma Linda University Medical Center (LLUMC, Loma Linda, CA, USA), the MD Anderson Cancer Center (MDACC, Houston, TX, USA), the Proton Medical Research Center (PMRC, Tsukuba, Japan), the Hyogo Ion Beam Medical Center (HIBMC, Tatsuno, Japan), and the National Cancer Center Hospital East (NCCHE, Chiba, Japan). Due to overlapping study periods from the same institutions reporting on the same disease stages, it is reasonable to assume that the same patients were analyzed in multiple reports. This was, however, not always obvious because follow-up periods and patient numbers differed. Therefore, it was decided to include all the published studies in this review. In instances where the studied patient cohort was identical (same number of patients, patient characteristics, and follow-up period), only the most recent study reporting on clinical outcome was selected. Study characteristics are summarized in Table 1 (early-stage NSCLC) and Table 2 (advanced-stage NSCLC).

**Table 1:** **Study descriptions early stage NSCLC**.

Study (characteristics); location	Beam, energy, technique	Chemotherapy	Fractionation schedule, TTD/*n* or fr/OTT	Patient selection criteria/risk factors	Stage distribution
**PROTON BEAM ONLY**					
Bush et al. (26) (prospective, *n* = 37); U.S. Loma Linda	Proton Photon + proton PSc	No	51 GyE/10 fr/2 weeks PBT Photons 45 Gy/25fr + protons, 28.8 GyE/16 fr/5 weeks	NSCLC stage I–IIIA; medically inoperable/refused surgery	Stage I, 27; Stage II, 2; Stage IIIA, 8
Shioyama et al. (27) (retrospective, *n* = 51); Japan, Tsukuba	Proton, 250 MeV, PSc (*n* = 33) Photon + proton (*n* = 18)	6 patients prior chemotherapy (all advanced stages)	Median 76 Gy (range 49-93 Gy), median fr dose 3.0 Gy (range 2–6 Gy), median OTT 43 days	NSCLC, medically inoperable/refused surgery	Stage IA/IB, 9/19; Stage IIA/IIB, 3/6; Stage IIIA/IIIB, 8; Stage IV, 1, recurrent disease, 5.
Bush et al. (14) (Phase II, *n* = 68); U.S. Loma Linda	Proton, PSc	No	51 GyE/10 fr/2 weeks (*n* = 22); 60 GyE/10 fr/2 weeks (*n* = 46)	NSCLC stage I, medically inoperable (*n* = 63)/refused surgery (*n* = 5)	Stage IA, 29; Stage IB, 39
Nihei et al. (15) (Retrospective, *n* = 37); Japan, Chiba	Proton, 150 or 190 MeV, PSc	No	70 GyE/20 fr/4–5 weeks (*n* = 3); 80 GyE/20 fr/4–5 weeks (*n* = 17); 88 GyE/20 fr/4–5 weeks (*n* = 16); 94 GyE/20 fr/4–5 weeks (*n* = 1)	NSCLC stage I; medically inoperable (*n* = 23)/refused surgery (*n* = 14), tumor size ≤ 5 cm, pO_2_ ≥ 60 torr, Zubrad PS 0-2	Stage IA, 17; Stage IB, 20
Hata et al. (16) (prospective, *n* = 21), Japan, Tsukuba	Proton, 155-200 MeV, PSc	No	50 GyE/10 fr/2 weeks (*n* = 3); 60 GyE/10 fr/2 weeks (*n* = 18)	NSCLC stage I, medically inoperable (*n* = 9)/refused surgery (*n* = 12), ECOG PS 0–2, no previous RT or chemotherapy for NSCLC	Stage IA, 11; Stage IB 10
Nakayama et al. (17) (retrospective, *n* = 55) (58 tumors) Japan, Tsukuba	Proton, 155–250 MeV, PSc	No	Peripheral tumors 66 GyE/10 fr/2 weeks (*n* = 41), central tumors 72.6 GyE/22 fr/4.5 weeks (*n* = 17)	NSCLC stage I; EORTC PS 0–2, medically inoperable (*n* = 52)/refused surgery (*n* = 3), Exclusion: pleural effusion, tumor close to stomach/esophagus	Stage IA, 30; Stage IB, 28
Bush et al. (18) (Phase 2, *n* = 111); U.S. Loma Linda	Proton, PSc	No	Dose escalation during study, 51 GyE/10 fr/2 weeks (*n* = 29), 60 GyE/10 fr/2 weeks (*n* = 56), 70 GyE/10 fr/2 weeks (*n* = 26)	NSCLC stage I, medically inoperable/refused surgery	Stage IA, 47; Stage IB, 64
Kanemoto et al. (19) (Retrospective, *n* = 74, 80 tumors), Japan, Tsukuba	Proton, 155–250 MeV, PSc	No	Peripheral tumors 66 GyE/10–12 fr/2–2.5 weeks (*n* = 59), central tumors 72.6 GyE/22 fr/4.5 weeks (*n* = 21)	NSCLC stage I	Stage IA, 59; Stage IB, 21
**C-ION (COMBINED WITH PROTON BEAM)**					
Iwata et al. (24) (retrospective, *n* = 80), Japan, Hyogo	Proton 150 MeV, PSc C-ion 320 MeV, PSc	No	Proton: 80 GyE/20 fr/4 weeks (*n* = 20), 60 GyE/10 fr/2 weeks (*n* = 37), C-ion: 52.8 GyE/4 fr/1 week (*n* = 23)	NSCLC stage I, medically inoperable (*n* = 47)/refused surgery (*n* = 43), WHO PS 0–2, no history lung cancer or previous chest RT/chemo	Stage IA, 42; Stage IB, 38
Iwata et al. (25) {retrospective [partially subgroup of cohort Iwata (24)], *n* = 70}, Japan, Hyogo	Proton 150 MeV, PSc C-ion 320 MeV, PSc	No	Proton: 80 GyE/20 fr/4 weeks (*n* = 14), 60 GyE/10 fr/2 weeks (*n* = 20), 66 GyE/10 fr/2 weeks (*n* = 8), 70.2 GyE/26 fr/5.5 weeks (*n* = 1) C-ion: 52.8 GyE/4 fr/1 week (*n* = 16), 66 GyE/10 fr/2 weeks (*n* = 8), 68.4 GyE/9 fr/2 weeks (*n* = 3)	NSCLC cT2a/T2bN0M0, medically inoperable (*n* = 40)/refused surgery (*n* = 30), WHO PS 0–2, no history lung cancer or previous chest RT/chemotherapy	Stage IB, 47; Stage IIA, 23
Miyamoto et al. (20) (phase I/II, *n* = 81, 82 tumors), Japan, Chiba	C-ion, 290–350–400 MeV, PSc	No	59.4–95.4 GyE/18 fr/6 weeks (*n* = 48), 68.4–79.2 GyE/9 fr/3 weeks (*n* = 34)	Inoperable NSCLC stage I, WHO PS 0–2, no history of RT to target, no prior chemotherapy <4 weeks	Stage IA, 41; Stage IB, 41
Miyamoto et al. (21) (phase II, *n* = 79), Japan, Chiba	C-ion, 290–350–400 MeV, PSc	No	52.8 GyE/4 fr/1 week (IA) 60 GyE/4 fr/1 week (IB)	NSCLC stage I, medically inoperable/refused surgery, WHO PS 0-2, no history of RT to target, no prior chemotherapy <4 weeks	Stage IA, 42; Stage IB, 37
Miyamoto et al. (22) (phase II, *n* = 50, 51 tumors), Japan, Chiba	C-ion, 290–350–400 MeV, PSc	No	72 GyE/9 fr/3 weeks	Peripheral NSCLC Stage I, WHO PS 0-2, no history of RT to target, no prior chemotherapy <4 weeks	Stage IA, 30; Stage IB, 21
Sugane et al. (23) (phase II, *n* = 28, 29 tumors), Japan, Chiba	C-ion, 290–350–400 MeV, PSc	No	72.0 GyE/9 fr/3 weeks (all, 1999–2000,*n* = 12), 60.0 GyE/4 fr/1 week (IA, *n* = 11), 52.8 GyE/4 fr/1 week (IB, *n* = 6)	NSCLC stage I; elderly, aged ≥80 years	Stage IA, 12; Stage IB, 17

*ECOG, Eastern Cooperative Oncology Group; EORTC, European Organization for Research and Treatment of Cancer; OTT, overall treatment time; PS, performance score; PSc, passive scattering; RT, radiotherapy; TTD, total tumor dose; WHO, World Health Organization*.

**Table 2:** **Study descriptions advanced-stage NSCLC**.

Study (characteristics); location	Beam, energy, technique	Chemotherapy	Fractionation schedule, TTD/*n* or fr/OTT	Patient selection criteria/risk factors	Stage distribution
**PROTON BEAM ONLY**					
Bush et al. (26) (prospective, *n* = 37); U.S. Loma Linda	Proton Photon + proton PSc	No	51 GyE/10 fr/2 weeks Photons, 45 Gy/25 fr + protons, 28.8 GyE/16 fr/5 weeks	NSCLC stage I–IIIA; medically inoperable/refused surgery	Stage I, 27; Stage II, 2; Stage IIIA, 8
Shioyama et al. (27) (retrospective, *n* = 51); Japan, Tsukuba	Proton, 250 MeV, PSc (*n* = 33) Photon + proton (*n* = 18)	6 patients prior chemotherapy (all advanced stages)	Median TD 76 Gy (range 49–93 Gy), median fr dose 3.0 Gy (range 2-6 Gy), median OTT 43 days	NSCLC, medically inoperable/refused surgery	Stage IA/IB, 9/19; Stage IIA/IIB, 3/6; Stage IIIA/IIIB, 8; Stage IV, 1, recurrent disease, 5.
Chang et al. (32) (phase II, *n* = 44), U.S., Houston	Proton, PSc	Concurrent, weekly carboplatin (2AUC) + paclitaxel (50 mg/m^2^) (*n* = 44). (neo) Adjuvant chemotherapy allowed (*n* = 19)	74 GyE/37fr/7.5 weeks	NSCLC Stage III, Unresectable/medically inoperable, KPS 70-100, weight loss not >10% during <6months before diagnosis	Stage IIIA, 21; Stage IIIB, 23
Nakayama et al. (31) (retrospective, *n* = 35), Japan, Tsukuba	Proton, 155-250 MeV, PSc	No	77 GyE/35 fr/7 weeks (*n* = 13), 83.6 GyE/38 fr/7.5 weeks (*n* = 7), 72.6 GyE/22 fr/4.5 weeks (*n* = 6), 74 GyE/37 fr/7.5 weeks (*n* = 3), other (*n* = 6)	NSCLC Stage II/III, medically inoperable/refused surgery, EORTC PS 0–2, unsuitable for/refusal chemotherapy. Exclusion: pleural effusion, tumor close to stomach/esophagus	Stage IIA, 2; IIB, 3; Stage IIIA, 12, Stage IIIB, 18
Xiang et al. (34) (prospective, *n* = 84), U.S. Houston	Proton, PSc	Concurrent, weekly carboplatin (2AUC) + paclitaxel (50mg/m^2)^ (*n* = 84). (neo) Adjuvant chemotherapy allowed (*n* = 22)	74 GyE/37/7.5 weeks	Stage III NSCLC, unresectable, availability of pre- and post-treatment PET-CT images	NR
Oshiro et al. (30) (retrospective, *n* = 57), Japan, Tsukuba	Proton, 200 MeV, PSc	No concurrent chemotherapy, induction chemotherapy: *n* = 14	Median 74 GyE (50-84.5 GyE), median fr dose 2.0 GyE (2.0–6.6 GyE)	Stage III NSCLC	Stage IIIA, 24; Stage IIIB, 33

*AUC, area under the curve; EORTC, European Organization for Research and Treatment of Cancer; KPS, Karnofsky Performance Status; NR, not reported; PS, performance score; PSc, passive scattering; TTD, total tumor dose*.

#### Early-stage NSCLC

##### Study descriptions

Fourteen studies investigated particle therapy for early-stage (stage I or II) NSCLC. The patients included were either medically inoperable or refused surgery, since surgery is still regarded as the first choice for treatment of Stage I NSCLC (11–13). Six studies reported the outcome of patients treated with protons only (14–19), four used carbon ions only (20–23), two studies reported outcome for both carbon ions and protons (24, 25), and two studies treated patients with a combination of photons and protons (26, 27). The most recent study is that of Kanemoto et al. (19). The researchers retrospectively evaluated disease control rates after high-dose proton beam therapy (PBT) in 74 patients with stage I NSCLC, with tumor doses of 66 Gy-equivalent (GyE) in 10–12 fractions for peripheral tumors (74% of all tumors) and 72.6 GyE in 22 fractions for centrally located tumors. Another retrospective study from the same center (with different investigators), evaluated the outcome of PBT for 55 medically inoperable patients with stage I NSCLC, using the same total dose and fractionation (17). The study periods of both studies overlap, making it reasonable to assume that the patients included in the study by Nakayama et al. (17) were also assessed by the Kanemoto group (19).

In both studies by Iwata et al. (24, 25), early-stage NSCLC patients were treated with either carbon ions or protons. In their first report, the authors reported the outcome for 80 stage I NSCLC patients treated with 60–80 GyE protons or 52.8 GyE carbon ions during the period 2003–2007 (24). After restaging based on the AJCC seventh edition of the TNM classification and a longer inclusion and follow-up period (median 51 months), they analyzed the outcome for 70 cT2a/2bN0M0 NSCLC patients in a second study (25). Some patients in the cohort were, therefore, included in both the studies. The choice of particle type was based on beam availability (during part of the study, only proton beams were available), and if both particle types were available, a choice was made after comparison of dose–volume histograms for both modalities.

The American group of Bush et al. (14, 18, 26) has performed three studies on proton beam irradiation for early-stage NSCLC in patients with contraindications to surgery or patients refusing surgery. In their first study, they treated 19 patients with insufficient pulmonary reserve or severe cardiac dysfunction with 51 GyE of PBT to the gross tumor volume (GTV). In 18 patients with sufficient cardiopulmonary function, 45 Gy in 1.8 Gy fractions was delivered with photon irradiation, followed by a proton beam boost to the GTV of 28.8 GyE in 1.8 GyE fractions. In 2004 and 2013, updates of the first trial were published, focusing on stage I NSCLC patients treated with proton therapy only (14, 18). The study published in 2013 investigated the outcome of 111 early-stage NSCLC patients, with dose escalation from 51 GyE in 10 fractions to 70 GyE in 10 fractions during the course of the study (18). Nihei et al. (15) treated 37 stage I patients with PBT in Chiba, Japan. Ten of the included patients were enrolled in an earlier dose escalation study and all patients received 70–94 GyE in 20 fractions (fraction dose 3.5–4.7 GyE).

The use of carbon ions for early-stage NSCLC was investigated in four phase II studies by Japanese researchers in a similar study population (20–23). Sugane et al. (23) focused on patients of 80 years or older within the study cohorts of Miyamoto et al. (21, 22). The patients received 52.8 GyE (stage IA) or 60 GyE (stage IB) in four fractions during 1 week, or (if treated prior to 2000) 72 GyE in 9 fractions over 3 weeks.

##### Overall survival and local control

The outcome of the selected studies for early-stage NSCLC is summarized in Table 3. The retrospective study by Nakayama et al. (17) observed 2-year OS and local control rates of 97.8 and 97.0%, respectively. However, the study had a relatively short follow-up period (median 17.7 months). No difference in local recurrence between tumors located centrally or peripherally in the lung was observed. Kanemoto et al. (19) reported a 3-year OS of 76.7% and a 3-year local control rate of 81.8% (stage IA 86.2%, stage IB 67%) with a median follow-up period of 31.0 months. The 3-year local control rate was significantly better for peripherally located tumors: 88.4 versus 63.9% for centrally located tumors.

**Table 3:** **Study outcomes early stage NSCLC**.

Study (characteristics); location	FU	OS	LC	DM	PFS	Toxicity
**PROTON BEAM ONLY**						
Bush et al. (26) (prospective, *n* = 35); U.S. Loma Linda	Median 14 months (range 3–45)	2 years 31% (Stage I: 39%)	2 years 87%	14%^a^	2 years 63% (Stage I: 86%)	G2 RP: 5.7%
Shioyama et al. (27) (retrospective, *n* = 51); Japan, Tsukuba	Median 30 months (range 18–153)	2 years 62%, 5 years 29% 2 years Stage I/II: 55%, Stage IA 88%, IB 47% 5 years Stage I/II 23%, IA 70%, IB 16%	5 years 57% (Stage IA 89%, IB 39%)	16%^a^	5 years 37% (Stage IA 89%, IB 17%)	Acute: lung tox ≤G1: 92%, G2: 6%, G3: 2%
Bush et al. (14) (Phase II, *n* = 68); U.S. Loma Linda	At least 12 months	3 years 44%: 51 GyE: 27% 60 GyE: 55%	3 years 74% (Stage IA: 87%, IB: 49%)	3 years 31%	3 years 72%	NR
Nihei et al. (15) (Retrospective, *n* = 37); Japan, Chiba	Median 24 months (range 3–62)	2 years 84%	1 years 91%, 2 years 80%	19%^a^	1 year 73%, 2 years 58%	Acute: G1: 84% (mostly dermatitis) No ≥grade 2 Late: lung toxicity (pneumonitis/pleural effusion): G2: 8% or G3: 8%
Hata et al. (16) (prospective, *n* = 21), Japan, Tsukuba	Median 25 months (range 10–54)	2 years 74% (Stage IA 100%, IB 47%)	2 years 95% (Stage IA 100%, IB 90%)	19%^a^	2 years 79% (Stage IA 89%, IB 70%)	Acute: hematological G1–2: 14%. Dermatitis G1: 19%, RP G2: 5% Late: G2: 10% (subcutaneous induration/myositis)
Nakayama et al. (17) (retrospective, *n* = 55) (58 tumors) Japan, Tsukuba	Median 17.7 months (range 1.4–53.3)	2 years 97.8%	2 years 97%	0%	2 years 88.7%, 3 years 78.9%	Acute: lung (pneumonitis) G1: 25.4%, G2: 3.6%, G3: 3.6% Late: rib fracture: 1.8%
Bush et al. (18) (phase 2, *n* = 111); U.S. Loma Linda	Median 48 months	4 years: 51 GyE 18%, 60 GyE 32%, 70 Gy 51% 4 years OS peripheral T1: 60%	4 years: 60 GyE 45%, 70 Gy 74% Peripheral T1: 96%	4 years peripheral T1: 81%	NR	Rib fractures: 3.6%, no other ≥G2 adverse events
Kanemoto et al. (19) (retrospective, *n* = 74, 80 tumors), Japan, Tsukuba	Median 31 months (range 7.3–104.3)	3 years 76.7%, 5 years 65.8%	3 years 81.8%, 5 years 81.8% 3 years Stage IA 86.2%, IB 67%	NR	3 years 58.6%, 5 years 52.5%	Acute: G2 skin: 2.5%, G2 esophagitis: 1.3%, G3 pneumonitis: 1.3% Late: G3 RP: 1.3%, G3 skin ulcer 1.3%, G4 rib fracture 13.8%
**C-ION (COMBINED WITH PROTON BEAM)**						
Iwata et al. (24) (retrospective, *n* = 80), Japan, Hyogo	Median 30.5 months (range 18–66)	3 years 75% (Stage IA 74%, IB 76%) 3 years OS by dose: 80 GyE 90%, 60 GyE 61%, 52.8 GyE 86%	3 years 82% (Stage IA 87%, IB 77%) 3 years LC by dose: 80 GyE 83%, 60 GyE 81%, 52.8 GyE 86%	16%^a^	3 years 54% (Stage IA 67%, IB 46%)	Lung toxicity: G2 RP: 11%, G3 RP: 1.3% Skin: dermatitis G2/3: 16% G2 rib fracture: 23%
Iwata et al. (25) {retrospective [partially subgroup of cohort Iwata (24)], *n* = 70}, Japan, Hyogo	Median 51 months (range 24–103)	4 years 58% (T2a 53%, T2b 67%)	4 years 75% (T2a 70%, T2b 84%)	20%^a^	4 years 46% (T2a 43%, T2b 52%)	G2/3 radiation pneumonitis 3%, G2/3/4 dermatitis 7%, Rib fracture G2 27%, soft tissue fibrosis G2 6%
		Operable patients (*n* = 30): 4 years 72%	Operable patients (*n* = 30): 4 years 73%		Operable patients (*n* = 30): 4 years 46%	
Miyamoto et al. (20) (phase I/II, *n* = 81, 82 tumors), Japan, Chiba	Median 52.6 months	5 years 42% (Stage IA 64.4%, Stage IB 22%)	23% local recurrence at 6.2–27.2 months after start RT	38%	NR	Acute: lung G2: 6.2%, G3: 3.7% Late: lung G2: 1.2%
Miyamoto et al. (21) (phase II, *n* = 79), Japan, Chiba	Median 38.6 months (range 2.5–72.2)	5 years 45% (Stage IA: 62%, IB 25%)	5 years 90% (Stage IA 98%, IB 80%)	27%^a^	NR	Acute: lung G2: 1.3% Late: lung G2: 1.3% No G3 toxicity
Miyamoto et al. (22) (phase II, *n* = 50, 51 tumors), Japan, Chiba	Median 59.2 months (range 6–83)	5 years 50% (Stage IA 55.2%, IB 42.9%)	5 years 95%	28%^a^	NR	Acute: lung toxicity: G1: 2%, G2: 2%. Skin G1: 100% Late: lung G1: 96%, G2: 4%. Skin G1: 98%
Sugane et al. (23) (phase II, *n* = 28, 29 tumors), Japan, Chiba	NR	2 years 64.3%, 5 years 30.7% (Stage IB 21.2%)	2 and 5 years: 95.8% (Stage IA 100%, IB 91.7%). Tumor >4 cm: 5 years LC 80%	NR		Acute: no lung toxicity. Skin G1: 96% Late: lung toxicity G1: 96%. Skin G1: 100%

*^a^Own calculation, time-point unknown*.

*DM, distant metastases; FU, follow-up; G, CTC Grade; LC, local control; NR, not reported; OS, overall survival; PFS, progression free survival; RP, radiation pneumonitis; RT, radiotherapy*.

In their first study in 2010, Iwata et al. (24) reported the outcome for 80 patients treated with either protons or carbon ions with a median follow-up of 30.5 months. They observed 3-year OS and local control rates of 75 and 82%, respectively. No significant difference in outcome was observed between the different treatment protocols. The authors also published the long-term outcome for cT2a and cT2b NSCLC patients (25). They reported a 4-year local control rate of 75%, and overall and progression-free survival rates of 58 and 46%, respectively. No significant differences were observed based on T stage. The survival of the operable patients was better than for the medically inoperable patients.

Studies reporting on the use of carbon ions for early-stage lung cancer demonstrate 5-year OS and local control rates of 45 and 90%, respectively (21), and of 30.7 and 95.8% (23) for patients aged 80 years or older.

##### Toxicity

The toxicity of PBT for early-stage NSCLC was found to be low, with the percentage of acute and late CTC grade ≥2 adverse events generally <10%. This is not a surprising outcome, since stereotactic body radiation therapy (SBRT) with photons for stage I NSCLC also has a generally favorable toxicity profile (13). Mainly for peripheral tumors, few treatment related complications have been reported (28). The treatment of central tumors located close to vulnerable mediastinal structures, however, can cause a higher rate of severe toxicity (29). The LungTech study (NCT01795521) is currently recruiting patients for a phase II trial of hypofractionated photon radiotherapy (60 Gy in 8 fractions given on alternating days) for centrally located tumors in patients with inoperable disease. Using PBT, Bush et al. (18) reported a low incidence of late rib fractures (4%), and in all of the cases, the tumor was located close to the chest wall. In contrast, other studies reported a relatively high incidence of Grade ≥ 2 rib fractures of 14–27% (19, 24, 25). In the experience of Iwata et al., the rib fractures mainly occurred in patients treated with only one beam portal (24, 25). These patients received a rib dose of 70–100% of the isocenter dose.

#### Locally advanced NSCLC

##### Study descriptions

Three studies have reported on the outcome of proton therapy for locally advanced NSCLC. The retrospective study of Oshiro et al. (30) evaluated the outcome of patients with stage III NSCLC after treatment with protons, without concurrent chemotherapy. The cohort consisted of 57 patients treated with a median dose of 74 GyE (range 50–85 GyE) in 2 GyE fractions (range 2–6.6 GyE). A respiratory gating system was used, and patients were immobilized with a body cast. Due to patient comorbidities, old age or patient refusal, concurrent chemotherapy was not administered. Fourteen patients received induction chemotherapy, however, without a tumor response. Nakayama et al. (31) reported on 35 stage II and III NSCLC patients treated in Tsukuba between 2001 and 2008 with protons to a median dose of 78.3 GyE. All patients were unsuitable for (*n* = 31) or refused chemotherapy or surgery (*n* = 4). Chang et al. (32) performed a phase II study at MD Anderson Cancer Center on the use of protons with concurrent chemotherapy for unresectable Stage III non-small-cell lung cancer. Forty-four stage III NSCLC patients were included and treated with 74 GyE using passively scattered protons, combined with weekly carboplatin and paclitaxel. Treatment simulation with four-dimensional CT was undertaken and an internal gross tumor volume (iGTV) was defined to account for tumor motion. Verification 4D CT scans were obtained in the third or fourth week of treatment. In case of significant changes regarding patient anatomy or tumor shrinkage, a new treatment plan was constructed [see Ref. (33)].

##### Overall survival and local control

After a median follow-up of 22.2 months (for surviving patients), Oshiro et al. (30) reported 1- and 2-year OS rates of 65.5 and 39.4%, respectively (Table 4). Progression-free survival and local control rates were 24.9% and 64.1% at 2 years, respectively. Distant metastases were most often the initial sites of recurrence. Induction chemotherapy did not have a significant effect on the OS.

**Table 4:** **Study outcomes advanced-stage NSCLC**.

Study (characteristics); location	FU	OS	LC	DM	PFS	Toxicity
**PROTON BEAM ONLY**						
Bush et al. (26) (prospective, *n* = 35); U.S. Loma Linda	Median 14 months (range 3–45)	2 years 31% (IIIA 13%)	2 years 87%	14%^a^	2 years 63% (IIIA 19%)	G2 RP: 5.7%
Shioyama et al. (27) (retrospective, *n* = 51); Japan, Tsukuba	Median 30 months (range 18–153)	2 years 62%, 5 years 29% 2 years Stage III/IV 62%, 5 years Stage III/IV 0%	5 years 57% (total group)	16%^a^	5 years 37% (total group)	Acute: lung tox ≤G1: 92%, G2: 6%, G3: 2%
Chang et al. (32) (phase II, *n* = 44), U.S., Houston	Median 19.7mnd (range 6.1–44.4)	1 year 86%	Local recurrence rate 20.5%	43%	1 year 63%	Acute: CT related G4 tox: 11.4%. G3 dermatitis: 11.4%, G3 esophagitis: 11.4%, G3 RP: 2.3%. No G4 toxicity Late: pulmonary/pleural fistula: 2.3%
Nakayama et al. (31) (retrospective, *n* = 35), Japan, Tsukuba	NR	1 year 81.8%, 2 years 58.9%	1 year 93.3%, 2 years 65.9%	20%	1 year 59.6%, 2 years 29.2%	Lung: G1: 25.7%, G2: 14.3%. G2 esophagitis: 2.9%. No ≥G3 toxicity
Xiang et al. (34) (prospective, *n* = 84), U.S. Houston	Median 19.2 months (6.1–52.4)	3 years 37.2%	2 years 83%	39% 3 years DMFS 35.4%	3 years 31.2%	NR
Oshiro et al. (30) (retrospective, *n* = 57), Japan, Tsukuba	Median 16.2 months	1 year 65.5%, 2 years 39.4%	1 year 79.1%, 2 years 64.1%	47%^a^	1 year 36.2%, 2 years 24.9%	Acute: lung (RP) G2: 7%, G3: 1.8%, G4: 1.8%, G5: 1.8%. Esophagitis G2: 1.8% Late: RP G2: 6.3%. Hemoptysis G5: 2.1%

*^a^Own calculation, time-point unknown*.

*DM, distant metastases; DMFS, distant metastasis free survival; FU, follow-up; G, CTC Grade; LC, local control; NR, not reported; OS, overall survival; PFS, progression free survival; RP, radiation pneumonitis; RT, radiotherapy*.

For the NSCLC patients included in the study by Nakayama et al. (31), the 2-year OS was 58.9% and the local progression-free survival at 2 years was 65.9%. Four patients (11.4%) developed a recurrence within the irradiated primary tumor volume, 10 patients (35%) developed a regional recurrence, and 7 patients (20%) developed distant metastases. Chang et al. (32) added concurrent chemotherapy to PBT and reported overall and progression-free survival of 86 and 63%, respectively, at 1 year, and a median OS time of 29.4 months. Again, distant metastases were the most frequent site of failure (19 patients, 43%). Only four patients (9.1%) had an isolated local recurrence. OS and progression-free survival (PFS) were better than those reported by Oshiro et al. (30), possibly due to the added effects of the concurrent chemotherapy or the pretreatment condition of the patients. While the patients included by Chang et al. (32) were fit enough to receive (concurrent) chemotherapy, most of those described by Oshiro et al. (30) were not (mostly due to old age and comorbidities). In an updated analysis of an expanded cohort of 84 patients treated at MD Anderson Cancer Center with concurrent chemotherapy and PBT for locally advanced NSCLC that assessed the correlation between post-treatment FDG uptake on PET/CT scan and clinical outcomes, including survival, Xiang et al. (34) reported a similar median survival time of 29.9 months. At 3 years, the local recurrence-free survival (LRFS) rate was 34.8%, distant metastasis-free survival (DMFS) rate was 35.4%, PFS rate was 31.2%, and the OS rate was 37.2%. Furthermore, post-treatment SUV was found on multivariate analysis to be independently prognostic for LRFS, DMFS, PFS, and OS (all *p* < 0.05).

##### Toxicity

Acute treatment toxicity was generally mild. Oshiro et al. (30) reported Grade ≥ 3 lung toxicity in three patients (5%): one patient had Grade 3 pneumonitis effectively treated with steroids, one patient had to discontinue treatment due to Grade 4 pneumonitis, and one patient died of pneumonitis during treatment (Grade 5). Both patients with the severe pneumonitis had severe preexisting interstitial pneumonitis prior to the diagnosis of NSCLC and, therefore, were deemed unsuitable for surgery or conventional (photon) radiotherapy. Three patients developed Grade ≥ 3 late toxicity: two patients with Grade 3 dyspnea, and one patient with Grade 5 hemoptysis (after repeated biopsy of the irradiated bronchus).

In the study by Chang et al. (32), toxicity was primarily related to the administered chemotherapy and consisted mainly of Grade 2 and 3 bone marrow suppression. A further five patients (11.4%) experienced Grade 3 esophagitis, one patient (2.3%) developed Grade 3 pneumonitis, and five patients (11.4%) had Grade 3 dermatitis. One patient developed a pulmonary/pleural fistula. No Grade 5 toxicity occurred. Toxicity was not reported in the expanded analysis by Xiang et al. Nakayama et al. (31) reported no Grade ≥ 3 toxicities.

### Proton therapy techniques and considerations for treatment planning and delivery

Protons can be delivered with two different radiation techniques: passive scattering proton therapy (PSPT) or PBS. In PSPT, the tumor volume is irradiated as a whole, using collimators and compensators for dose conformality. With PBS, the target volume is scanned spot-by-spot with a narrow proton beam, enabling intensity-modulated proton therapy (IMPT). Most institutions treating NSCLC with protons use the passive technique as it is the most widely available and moreover less sensitive to breathing motion than PBS.

Matney et al. (35) quantified and compared the effects of respiratory motion on clinically delivered IMRT and re-calculated PSPT plans for 20 stage II-IIIb NSCLC patients. For a respiratory motion of up to 17 mm, target coverage was maintained for both IMRT and PSPT. Only two of the studied comparative parameters, lung V5 and spinal cord Dmax, were statistically significantly better when using PSPT. The authors concluded that PSPT may not be more susceptible to respiratory motion than IMRT.

Passive scattering proton therapy also leads to a non-conformal dose distribution at the proximal edge of the field. Conformality is better for the PBS technique, but the interplay between the intrafractional tumor motion and the scanned proton beams can have detrimental effects on the dose distribution. This so-called “interplay effect” can result in severe under- or overdosage: a spot can be irradiated several times or not at all. Thus, the dose is not only inhomogeneous at the edges but also inside the target, and therefore, cannot be accounted for by simply adding a surrounding margin (36). This is crucial especially for full IMPT that, in contrast to the single field uniform dose (SFUD) technique, results in completely inhomogeneous dose distributions per field. To our knowledge, only the MD Anderson Cancer Center has started to treat NSCLC with PBS using the SFUD technique, limiting the treatment to a subset of tumors with motion amplitudes below 5 mm. Li et al. (37) investigated the extent of the interplay effect on IMPT delivery and concluded that for the specific system at MDACC, the this effect may not be a primary concern. Grassberger et al. (38) and Dowdell et al. (39) have reported that the magnitude of the interplay effect depends on patient parameters such as motion amplitude and beam delivery parameters such as spot size. Several techniques (rescanning, gating, tracking) trying to compensate for the interplay effect are topic of intensive research in several groups worldwide. For IMPT, the robustness and a geometrical tumor tracking method mitigating to motion were evaluated in 7 NSCLC patients treated to 45 GyE in 3 fractions (40). Patients had in total nine peripheral stage I lung tumors that were imaged with 4D-CT, and the combined dose distribution was summed using deformable image registration. Additionally, plans were constructed in which the proton beams were geometrically shifted without adjusting the beam energy. In 6 tumors showing a displacement of <1 cm, 97–100% of the GTV was covered by 95% of the prescribed dose. For the remaining three patients, this was 95, 82, and 51%, respectively; subsequently, the application of a geometrical tracking method improved this to 100, 98, and 97%, respectively. The authors concluded that the simple tracking method was valuable for improving GTV coverage. The practicalities and limitations of tracking and gating techniques in IMPT have also been reported by the group at Paul Scherrer Institute (41, 42).

Another key aspect is plan robustness, meaning the susceptibility of the nominal treatment plan due to uncertainties (e.g., setup uncertainties) or motion. Some treatment planning systems already allow the evaluation of plan robustness. Furthermore, consideration of robustness can not only be included in the treatment plan evaluation step but also in the plan optimization (robust optimization) (43–46). Usually, this will result in decreased quality of the nominal treatment plan in combination with smaller deviations between nominal and actual dose distribution under the influence of setup uncertainties and intra- and interfractional motion.

For PSPT, range and setup uncertainties can be taken into account by widening the aperture to ensure the lateral coverage in the presence of setup and/or in-patient target shifts. Furthermore, density changes lateral to the beam path, e.g., due to intrafractional motion, are mitigated by “smearing” the compensator. Range uncertainties are accounted for by increasing the nominal range. This method was investigated by several groups (9, 47) and is widely accepted in PSPT in the clinic (8, 48–50).

For PBS, no physical devices are used. Consequently, a different method needs to be applied to compensate for range and setup uncertainties. Laterally, an increased treatment area adding a margin to the tumor volume is applicable. For range uncertainties, a more sophisticated approach is the use of field-specific PTVs that take into account the influences of range uncertainty for each treatment field separately (10, 51, 52). In comparison to a uniform margin extension, this leads to a PTV that can compensate for the specific requirements in proton therapy and thus preserves the target coverage in the presence of uncertainties.

### (*In silico*) Comparative Studies

Various institutions employing particle therapy or in the process of setting up the facility have published findings on comparative *in silico* planning studies using different photon and proton delivery techniques. Kase et al. (53) compared PSPT with IMPT in a variety of primary tumor sites, including NSCLC. IMPT resulted in lower dose to organs at risk, specifically, the high dose to the skin, the D20 to the normal lung, and the spinal cord.

#### 

##### Early-stage NSCLC

Wang and colleagues (54) from the Proton Medical Research Center compared 3D-CRT to PSPT in 24 patients with peripheral stage I NSCLC. Two to four proton beam ports were used and irradiation was applied at end exhalation. Photons were delivered using 5 to 7 coplanar ports covering the same clinical and planning target volume (CTV and PTV, respectively). The prescribed dose was 66 GyE in 10 fractions at the isocenter. While the 90% isodose line covered >99% of the CTV for both treatment modalities, the 95% isodose line covered only 86.4% of the CTV for proton plans and 43.2% for 3D-CRT plans. Organ at risk (OAR) doses, specifically, lungs, heart, esophagus, and spinal cord, were significantly lower for the proton beam technique.

The Mayo Clinic Group (55) generated treatment plans for eight stage I NSCLC patients with peripheral lung nodules using photon SBRT, and one-, two-, and three-field passively scattered or actively scanned proton beams. For SBRT (3 × 20 Gy), 10 or more non-coplanar beams were manually selected to achieve optimal PTV coverage while minimizing dose to the OARs. Plans were normalized to isocenter with the prescription isodose line covering 95% or more of the PTV. For proton beam treatment, beam direction was manually optimized to maximize access to the tumor, while minimizing exposure to OARs and adjacent normal tissues. Proton beam plans demonstrated significantly lower maximum and higher minimum PTV doses compared with SBRT. With the exception of the three-field actively scanned approach, the maximum dose 2 cm from the PTV was significantly higher with proton beams. The doses to OARs (lungs, spinal cord, heart, bronchial tree, esophagus, skin, and ribs) were generally lower with protons than with photons. Using actively scanned beams, the maximum dose to the PTV, V30Gy, and the dose to any tissue 2 cm from the PTV decreased, while the minimum dose to the PTV increased.

Similarly, Kadoya et al. (56) studied 21 patients with peripheral stage I NSCLC, delivering a dose of 66 GyE in 10 fractions during maximal expiration using SBRT (7–8 non-coplanar 4-MV photon beams) or PSPT (2–3 directions). While the dose to the PTV was non-significantly different, the dose to the lung was significantly lower with the PSPT technique. The authors concluded that PSPT may be advantageous for large or multiple PTVs.

For stage I NSCLC, PBT may provide the greatest dosimetric and clinical benefit for patients with centrally located tumors given the higher reported toxicity when delivering SBRT for tumors in this region. For centrally and superiorly located stage I NSCLC, Register et al. (57) compared SBRT with PSPT and IMPT. SBRT was prescribed to 50 Gy in 12.5-Gy fractions, normalized such that 95% of the PTV received 100% of the prescribed dose. Each PSPT plan was created with three to four coplanar beam angles in an attempt to minimize the exit dose into the lung parenchyma. The same beam angels were used for generating the IMPT plans. Only 6 of 15 photon SBRT plans satisfied PTV coverage and all normal tissue dose constraints, compared to 12 PSPT and 14 IMPT plans.

A significant reduction in the normal tissue dose by passively scattered protons (66 GyE) compared to 3D-CRT or IMRT photon treatment (66 Gy) was reported by Chang et al. (58) for stage I and stage IIIA/B NSCLC patients. For early-stage NSCLC (*n* = 10), the DVH comparison revealed a reduction in dose to the ipsilateral and contralateral lungs, heart, spinal cord, and esophagus with protons. For locally advanced patients (*n* = 15) in all cases, the doses to lung, heart, esophagus, spinal cord, and integral dose were lower with proton therapy than both 3D-CRT and IMRT photon modalities. This dosimetric benefits of protons over photons persisted even when escalating the PBT doses to 87.5 GyE for stage I NSCLC and 74 GyE for stage III NSCLC compared with 66 Gy for all photon plans.

An *in silico* planning study comparing modern photon techniques with protons and carbon ions in 25 stage I NSCLC patients is currently being conducted by the ROCOCO consortium (NCT02038413).

##### Locally advanced NSCLC

**Figure 1:**
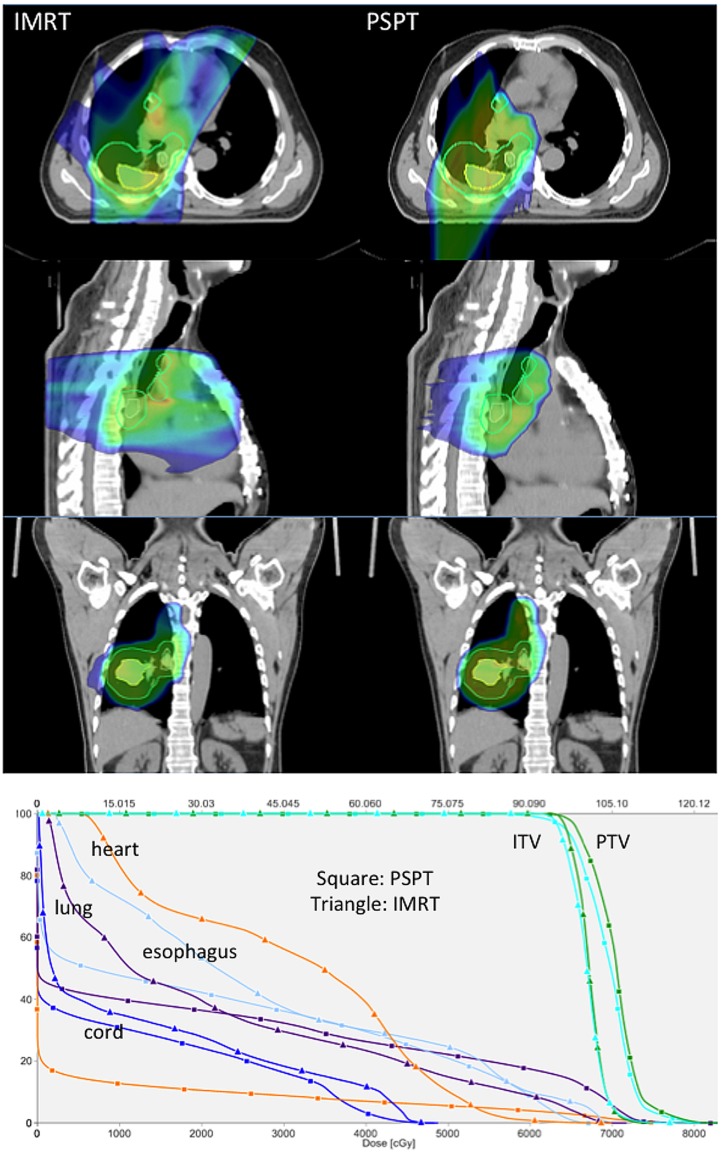
**Treatment planning images and dose–volume histogram comparison (Hospital of the University of Pennsylvania)**. Representative treatment planning images for a patient with locally advanced non-small cell lung cancer with a right lower lobe primary tumor (iGTV primary tumor depicted as a yellow contour) and multi-station mediastinal nodal metastasis (iGTV nodal metastasis depicted as a yellow contour). The composite PTV is depicted as a cyan contour. Comparative plans for IMRT (left) and proton beam therapy (right) are depicted in the axial planes (top row), sagittal planes (middle row), and coronal planes (bottom row) delivering 66.6 Gy (IMRT) or 66.6 GyE (proton therapy) in 37 fractions. Dose color wash coding: blue = 50% to red = global max above 100%. Representative dose–volume histograms for the same patient are also depicted showing dose to target volumes of PTV (green) and ITV (cyan) and dose to normal structures of heart (orange), total lung minus GTV (purple), esophagus (light blue), and spinal cord (dark blue) for IMRT (triangle) and proton (square) plans.

**Figure 2:**
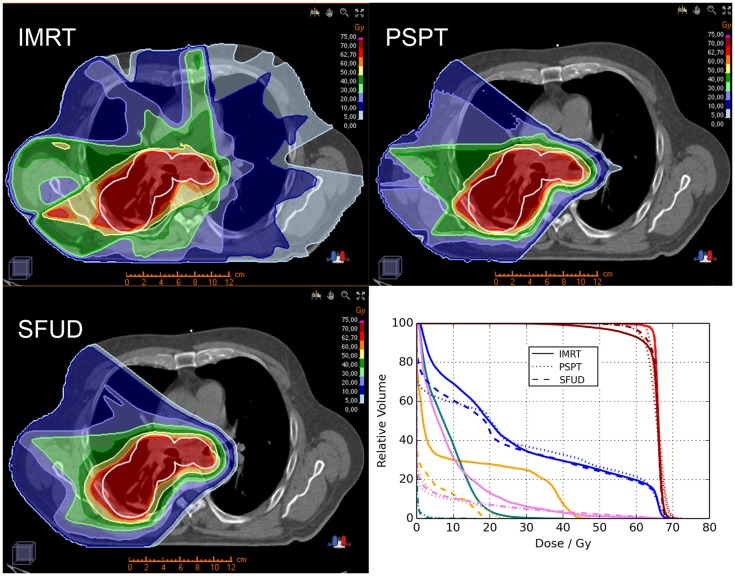
**Treatment plan comparison between IMRT (left upper row), PSPT (right upper row) and PBS using SFUD (left lower row) in an advanced-stage NSCLC patient (OncoRay, Dresden)**. The planning target volume (PTV) is represented by the white contour. Furthermore a dose–volume histogram (DVH; right lower row) analysis is illustrated for the three treatment techniques showing the clinical target volume (CTV; red), PTV (dark red), ipsilateral lung (blue), spinal cord (orange), heart (pink), and contralateral lung (turquois).

Proton beam therapy may be advantageous for locally advanced NSCLC patients delivering either the same dose with less toxicity or a higher dose under isotoxic conditions. Representative images showing plan comparisons for patients with locally advanced NSCLC are shown in Figures 1 and 2. In the first, PSPT spares significant volumes of heart, esophagus, and lung relative to IMRT. In the latter comparing IMRT, PSPT, and PBS using SFUD, the dose to the heart, spinal cord, and contralateral lung is significantly reduced using (advanced) proton techniques.

The MD Anderson Cancer Center conducted a prospective phase II clinical study combining chemotherapy with PSPT in 44 unresectable NSCLC patients (details and outcome of the study described earlier (32). Koay et al. (33) published an analysis for the nine out of 44 patients for whom the plans were adapted during the treatment course. While the authors observed profound differences in the dose to the esophagus and spinal cord, no statistical comparison of the adapted versus non-adapted patients was performed. For two of the nine patients, the internal CTV coverage would have decreased significantly without adaptation. Remarkably, the reported toxicity was higher among the patients treated with adaptive plans, although statistical comparison was again lacking. Treatment outcome was not significantly different between the treatment strategies (*p* > 0.05).

Zhang et al. (59) compared IMPT with PSPT and IMRT in 20 NSCLC patients with extensive stage IIIB disease. Using IMRT, the selected patients had no or borderline tolerance to IMRT at 60–63 Gy when keeping within the normal tissue dose constraints. IMPT succeeded in sparing more lung, heart, spinal cord, and esophagus when maintaining the prescribed dose. IMPT was even feasible when escalating the dose up to 83.5 Gy (mean maximum tolerated dose 74 Gy), whereas PSPT was limited by the esophagus dose constraint (74 Gy). The University of Florida Proton Therapy Institute (60) compared 3D-CRT with IMRT and PSPT in eight NSCLC patients. The target dose prescription was achieved in all patients using either of the techniques. Compared to the photon techniques, PSPT considerably reduced the dose to radiation-sensitive normal structures: normal lung V20Gy by median 29% for 3D-CRT and 26% for IMRT, mean lung dose by 33 and 31%, and V10Gy bone marrow by 30 and 27%, respectively. This may offer an advantage in patients undergoing sequential or concurrent radiochemotherapy. In a multicentric *in silico* clinical trial, the ROCOCO consortium compared photon (3D-CRT and IMRT) with proton (PSPT) treatment for stage IA-IIIB NSCLC patients delivering 70 Gy in 35 fractions (8). The integral dose for the photon techniques was higher than for the proton techniques, whereas the mean lung dose was lower for protons. For 10 patients, dose escalation to the primary tumor up to 87 Gy was feasible for all three modalities; however, the mean lung and integral doses were higher for photons than for protons.

## Conclusion

Proton beam delivery has evolved rapidly over the last decade, enabling highly conformal treatment delivery with the possibility of escalating the dose to the primary tumor while maintaining the dose to the normal tissues, or maintaining the dose to the target volume and reducing the dose to the organs at risk. Dosimetric advantages of protons over photons have been demonstrated across NSCLC stages of disease in reducing doses to critical organs at risk, including the lungs, heart, esophagus, and spinal cord. PBT has also been used clinically in an increasing number of prospective studies for both stage I NSCLC and locally advanced NSCLC with a generally more favorable toxicity profile than what has been reported with historical photon studies. Particle beam treatment may prove to provide its greatest clinical benefit compared with IMRT and photon treatment techniques for patients with pretreatment severe functional impairments (e.g., poor lung function, preexisting lung disease), when delivering escalating doses of radiation therapy or using radiation therapy as part of trimodality therapy combined with chemotherapy and surgery, and for definitive re-treatment of NSCLC patients with a local/locoregional recurrence following prior radiotherapy. From the *in silico* trials we have gathered, we can conclude that the dose distributions with IMPT are superior to those of the photon treatments and the passive scattering technique. Until now, PSPT has predominantly been used in the clinic instead of IMPT. It is likely that a more widespread introduction of IMPT can provide further improvement in lung cancer outcome and/or decreased toxicity, through increased precision and personalized treatment, provided that range uncertainties are well accounted for.

## Conflict of Interest Statement

The authors declare that the research was conducted in the absence of any commercial or financial relationships that could be construed as a potential conflict of interest.
